# HFpEF as systemic disease, insight from a diagnostic prediction model reminiscent of systemic inflammation and organ interaction in HFpEF patients

**DOI:** 10.1038/s41598-024-55996-5

**Published:** 2024-03-05

**Authors:** Rong Zhou, Yi-Yuan Xia, Zheng Li, Li-Da Wu, Yi Shi, Zhi-Yu Ling, Jun-Xia Zhang

**Affiliations:** 1 Department of Intensive Medicine, Qujing No. 1 Hospital, Qujing, 655000 Yunnan China; 2 Department of Cardiology, Nanjing First Hospital, Nanjing Medical University, Nanjing, 210006 Jiangsu China; 3 Department of Cardiology, The Second Affiliated Hospital of Chongqing Medical University, Chongqing, 404100 China

**Keywords:** Heart failure preserved ejection fraction, Inflammation, Organ interaction, Diagnostic prediction model, Nomogram, Biomarkers, Interventional cardiology, Cardiovascular diseases, Heart failure

## Abstract

Systemic inflammation and reciprocal organ interactions are associated with the pathophysiology of heart failure with preserved ejection fraction (HFpEF). However, the clinical value, especially the diagnositc prediction power of inflammation and extra-cardiac organ dysfunction for HfpEF is not explored. In this cross-sectional study, 1808 hospitalized patients from January 2014 to June 2022 in ChiHFpEF cohort were totally enrolled according to inclusion and exclusion criteria. A diagnostic model with markers from routine blood test as well as liver and renal dysfunction for HFpEF was developed using data from ChiHFpEF-cohort by logistic regression and assessed by receiver operating characteristic curve (ROC) and Brier score. Then, the model was validated by the tenfold cross-validation and presented as nomogram and a web-based online risk calculator as well. Multivariate and LASSO regression analysis revealed that age, hemoglobin, neutrophil to lymphocyte ratio, AST/ALT ratio, creatinine, uric acid, atrial fibrillation, and pulmonary hypertension were associated with HFpEF. The predictive model exhibited reasonably accurate discrimination (ROC, 0.753, 95% CI 0.732–0.772) and calibration (Brier score was 0.200). Subsequent internal validation showed good discrimination and calibration (AUC = 0.750, Brier score was 0.202). In additoin to participating in pathophysiology of HFpEF, inflammation and multi-organ interactions have diagnostic prediction value for HFpEF. Screening and optimizing biomarkers of inflammation and multi-organ interactions stand for a new field to improve noninvasive diagnostic tool for HFpEF.

## Introduction

Heart failure (HF) is the end-stage syndrome of diverse heart diseases. It is a rapidly growing and life-threatening public health problem as the aging of global population. The prevalence of HF is more than 64.3 million people around the world^1^, and approximately 50% of patients with HF have heart failure preserved ejection fraction (HFpEF)^2^. There are heterogeneities in both etiologies and phenotypes of HFpEF^3^. Thus far, the treatment of HFpEF still remains challenging. The DELIVER trial found that the sodium-glucose cotransporter 2 inhibitor dapagliflozin could reduce the risk of cardiovascular death and worsening of HF^4^. The EMPEROR-preserved study also found that treatment with the sodium-glucose cotransporter 2 inhibitor empagliflozin reduced the composite end point of cardiovascular death and hospitalization for HF by 21% in patients with HFpEF^5^.

The gold standard for diagnosis of HFpEF is right heart catheterization (RHC) followed by invasive exercise testing^6^. Considering the medical cost and invasiveness, RHC is not universally available. Recently, scoring systems have been developed to improve the diagnostic accuracy for HFpEF, and increasing evidences suggested invasive or non-invasive exercise testings in those with at least intermediate HFpEF probability^7^. The H_2_FPEF and HFA–PEFF noninvasive scoring systems for the diagnostic workup of HFpEF have been reported^8,9^, which are considered as reliable methods in diagnosis and treatment of cardiovascular diseases (CVDs)^10^. The H_2_FPEF score enables discrimination of HFpEF from noncardiac causes of dyspnea, relying on clinical characteristics (age > 60 years, obesity, atrial fibrillation, treatment with ≥ 2 antihypertensives) and echocardiographic measurements (E/E’ ratio > 9, pulmonary artery systolic pressure (PASP) > 35 mmHg)^8^. The HFA–PEFF score provides a new stepwise diagnostic process, including pre-test assessment, data from echocardiography and natriuretic peptides, and functional testings^9^.

Currently, systemic inflammation-induced microvascular endothelial dysfunction is deemed as one of the major contributors for the pathogenesis and progression of HFpEF^11,12^. Clusters of inflammation proteins were reported to mediate the coupling of comorbidity burden, right ventricular dysfunction and poor outcomes of HFpEF by increasing cardiomyocyte passive tension and aggravating aberrant myocardial collagen deposition^13,14^. In addition, local inflammation could lead the hypophosphorylation of giant sarcomeric protein titin by reducing nitric oxide and cyclic guanosine monophosphate availability, and increase myocardial stiffness and worsen diastolic function^15^. The neutrophil to lymphocyte ratio (NLR), a sensitive inflammatory marker, has gained recognition correlated with poor outcomes in CVDs^16^, and a significant elevation of NLR was observed in patients with HFpEF^17^. In addition, elevated uric acid (UA) has been considered as a marker of inflammatory cytokine activation^18^. It is common that patients with HFpEF have hyperuricemia, and several studies have revealed the correlation of elevated UA level with adverse outcomes of HFpEF^19–21^. Moreover, renal dysfunction^22^ and liver diseases^23,24^, were clinical co-morbidities for HFpEF. However, the clinical value, especially the diagnositc prediction power of inflammation and extra-cardiac organ dysfunction for HFpEF is not explored. We proposed a new diagnostic scoring systems with inflammatory markers and markers for extra-cardiac organ dysfunction in the study.

## Materials and methods

### Study design, data source

ChiHFpEF cohort is a prospective Study of HFpEF in Chinese Han patients with documented CVDs in Nanjing First Hospital, Nanjing Medical University (NCT05278026)^25^. In this study, 2967 patients in ChiHFpEF cohort were totally enrolled. According to inclusion and exclusion criteria, 1808 hospitalized patients from January 2014 to June 2022 with full recordings of routine blood test, liver and renal function test were included in the final analysis. This study was approved by the institutional research ethics committee of Nanjing First Hospital, Nanjing Medical University (KY20211011-04). This study was performed in accordance with the Declaration of Helsinki, and all patients signed the informed consent.

### Participants

#### Inclusion criteria

1. Patients aged > 18.
2. Patients with at least one following cardiovascular comorbidity: coronary heart disease, or hypertension, or diabetes mellitus.
3. LVEF ≥ 50%, determined by Simpson’s biplane method.

#### Exclusion criteria

1. Patients declined the N-Terminal pro B-type natriuretic peptide (NT-pro BNP) test.
2. Patients declined echocardiography or with LVEF < 50%.
3. Patients had severe hepatic impairment, including alanine aminotransferase (ALT) > 140U/L, or aspartate aminotransferase (AST) > 140U/L.
4. Patients refused to sign informed consent.
5. Patients with arrhythmia causing aberrant hemodynamics, chronic obstructive pulmonary disease, sleep apnea, aortic dissection, peripheral vascular diseases, pericardial diseases, myocarditis, hypertrophic cardiomyopathy, severe valvular heart diseases, cardiovascular neurosis, amyloidosis, costochondritis, shock, thyroid diseases and infectious diseases.

### Predictor variables

The candidate variables were extracted based on already published literature and expert consensus. We collected the data on demographics, and comorbidities from medical record. The following laboratory parameters were obtained: white blood cell count (WBC count), lymphocyte count, monocyte count, neutrophil count, eosinophil count, basophil count, hemoglobin (Hb), ALT, AST, blood urea nitrogen (BUN), creatinine (Cr), UA, total-cholesterol, triglycerides, high-density lipoprotein cholesterol (HDL-C), low-density lipoprotein cholesterol (LDL-C), Apolipoproteins A1 (Apo A1), Apolipoproteins B (Apo B), and lipoprotein A (Lpa). The following indices of echocardiography were collected: left atrium diameter (LAD), left ventricular diameter in diastole (LVDd), left ventricular diameter in systole (LVDs), end diastolic thickness of posterior left ventricular wall (LVPWD), interventricular septal thickness (IVSD), fractional shortening (FS), stroke volume (SV), LVEF, the maximum early transmitral flow velocity (Peak E velocity), the maximum early transmitral flow velocity in atrial systole (Peak A velocity), myocardial tissue velocity measured atthe septal and/or lateral mitral annulus (Peak E' velocity), the maximum myocardial tissue velocity measured at the mitral annulus in atrial systole (Peak A' velocity), E/A ratio, E'/A' ratio, and E/E' ratio. The laboratory tests, including routine blood test, renal and liver function tests, and NT-pro BNP level were measured on the next morning after admission. The echocardiography was performed by trained and qualified sonographers, and the quality of echocardiographic images was controlled by the Cardiovascular Imaging Laboratory of Nanjing First Hospital, Nanjing Medical University. The coronary heart disease was defined by coronary artery stenosis ≥ 50% on percutaneous coronary angiography or CT coronary imaging. The hypertension was defined by a systolic blood pressure ≥ 140 mmHg and/or diastolic blood pressure ≥ 90 mmHg measured three times on non-same day. The diabetes mellitus was defined as fasting plasma glucose ≥ 7.0 mmol/L, or random blood glucose ≥ 11.0 mmol/L, or a hemoglobin A1c ≥ 6.5 mg/dL. The NLR was defined as the ratio of neutrophil count to lymphocyte count. The AST/ALT ratio was defined as the ratio of AST to ALT. The atrial fibrillation (AF) was diagnosed by clinical history and electrocardiograph, and pulmonary hypertension (PH) was evaluated by PASP from the echocardiography^26,27^.

### Outcomes

HFpEF was diagnosed independently by two expert cardiologists according to the combinations of clinical manifestation, echocardiography and the NT-pro BNP, and any disagreement would be discussed. The diagnosis of HFpEF was made by the following criteria^6^:

1. Patients with orthopnoea, paroxysmal nocturnal dyspnoea, fatigue, pulmonary rales, bilateral ankle oedema, or other clinical signs and symptoms.
2. The LVEF ≥ 50%.
3. NT-pro BNP ≥ 125 pg/mL with sinus rhythm and ≥ 450 pg/mL with AF.
4. To fulfil at least one following additional criterion revealing cardiac functional and structural alterations: LAD > 40 mm; E/E’ ≥ 13, E’/A’ < 1.

### Handling of missing data

Of all 44 candidate variables, 31 variables contained missing values. It was not randomly occurred for the missing values of peak velocity in the echocardiography because there were the patients with AF included^28^. Therefore, we dismissed these missing values of peak velocity. The residual missing values ranged from 1 (0.06%) missing value for age to 169 (9.35%) missing values for Apo B. In previous studies, missing values were imputed by applying multiple imputation^8,29^. Cummings’ study suggested that multiple imputation might be not superior to other interpolation methods when the proportion of missing data was less than 10%^30^. Hence, for these low missing values, we imputed them via the EM algorithm to minimize the bias in our study.

### Statistical analysis

#### Variable selection and development of model

Continuous variables were reported as mean and SD or median and interquartile ranges according to their distribution, and categorical variables were summarized as the proportions within each category. First, univariate regression analysis was performed to screen the significant candidate predictors for HFpEF using SPSS software (IBM SPSS Statistics for Windows, Version 21.0. Armonk, NY: IBM Corp.). Continuous variables were compared using the t-test or Mann–Whitney U test, and categorical variables were compared using the Chi-squared test or Fisher's exact test. A *p*-value of less than 0.05 was considered statistically significant. Second, based on significant candidate predictors obtaining from univariate analysis, multivariate and least absolute shrinkage and selection operator (LASSO) regression analyses were applied, respectively, and the intersection was taken for screening optimal predictors for HFpEF. The stepwise forwards method was performed in multivariate regression analysis by using SPSS. The LASSO regression analysis was performed by using “glmnet” package in R software (version 4.1.2; http://www.r-project.org/). Third, based on these optimal predictors, the final model was refitting by using a logistic regression model.

#### Evaluation and validation of model

**Figure 1:**
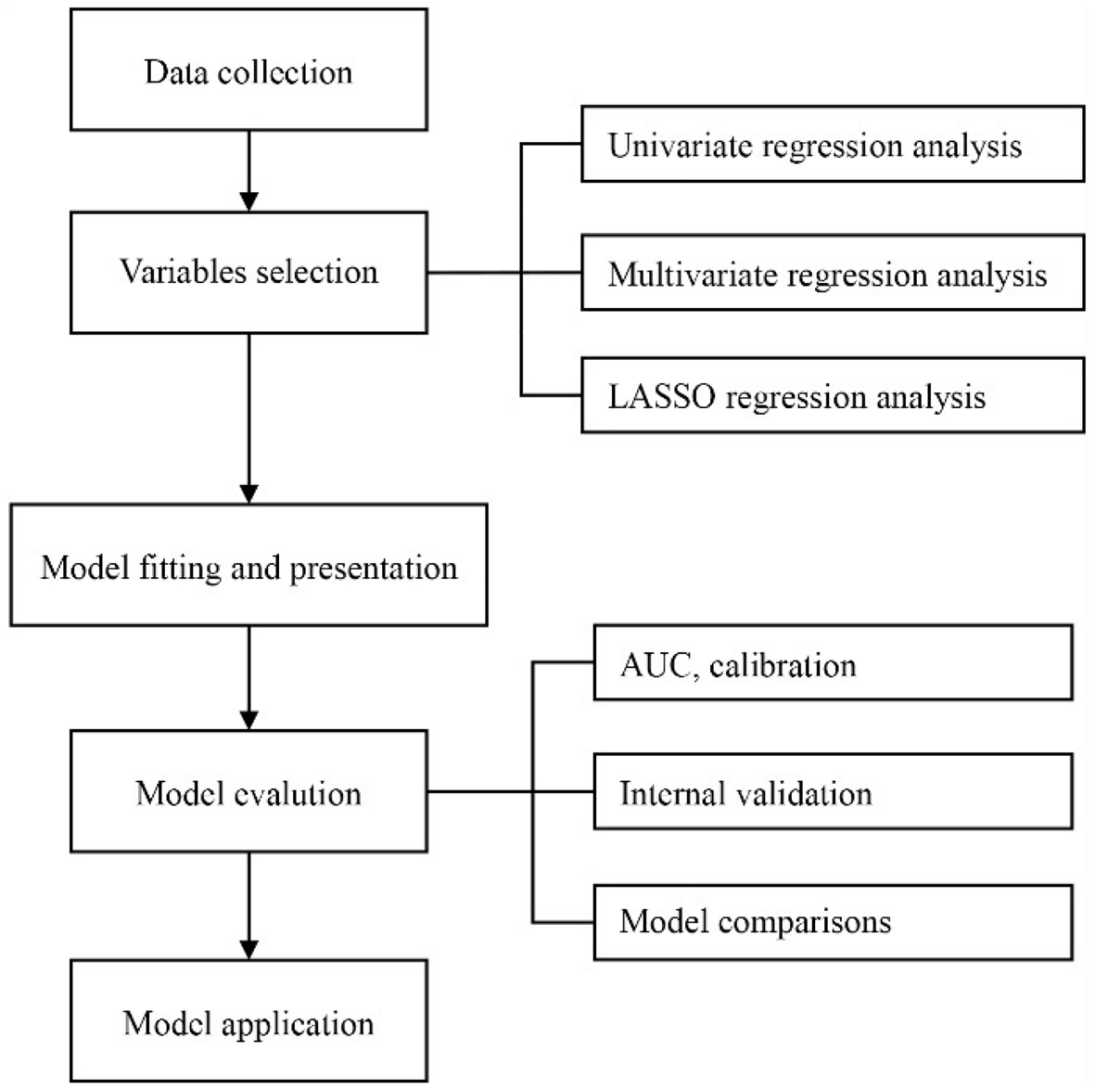
The flowchart of predictive model construction.

At the stage of model evaluation, the predictive ability for patients with HFpEF was calculated. The discrimination of model was evaluated by the area under the curve (AUC)^31^. Receiver operating characteristic curve (ROC) was plotted by using MedCalc software, and AUC and cut-off points for sensitivity or specificity levels were obtained as well. The calibration of model was assessed by the Brier score^31^. The calibration curve was plotted by using R “rms” package. The potential multicollinarity of model was diagnosed using the variance inflation factor (VIF) by the R “car” package^32^. Internal validation of model was performed using tenfold cross-validation. The discrimination and calibration of internal validation were determined by the mean of AUC and Brier score. The tenfold cross-validated calibration plot was used to exam graphically the model calibration. In order to evaluate the importance of each predictor in terms of prediction for HFpEF, the new regression model was refitted by removing any one predictor in the original model. Then, the predictive ability of each new model was calculated. The Delong method and likelihood ratio test were applied for comparing predictive ability, and fitting ability between original model and each new model, respectively. In addition, a nomogram was generated using R “rms” package, and a web-based online risk calculator using the R “shiny” package was designed as well. Figure 1 showed the flowchart of predictive model construction.

## Results

### Patient characteristics

**Figure 2:**
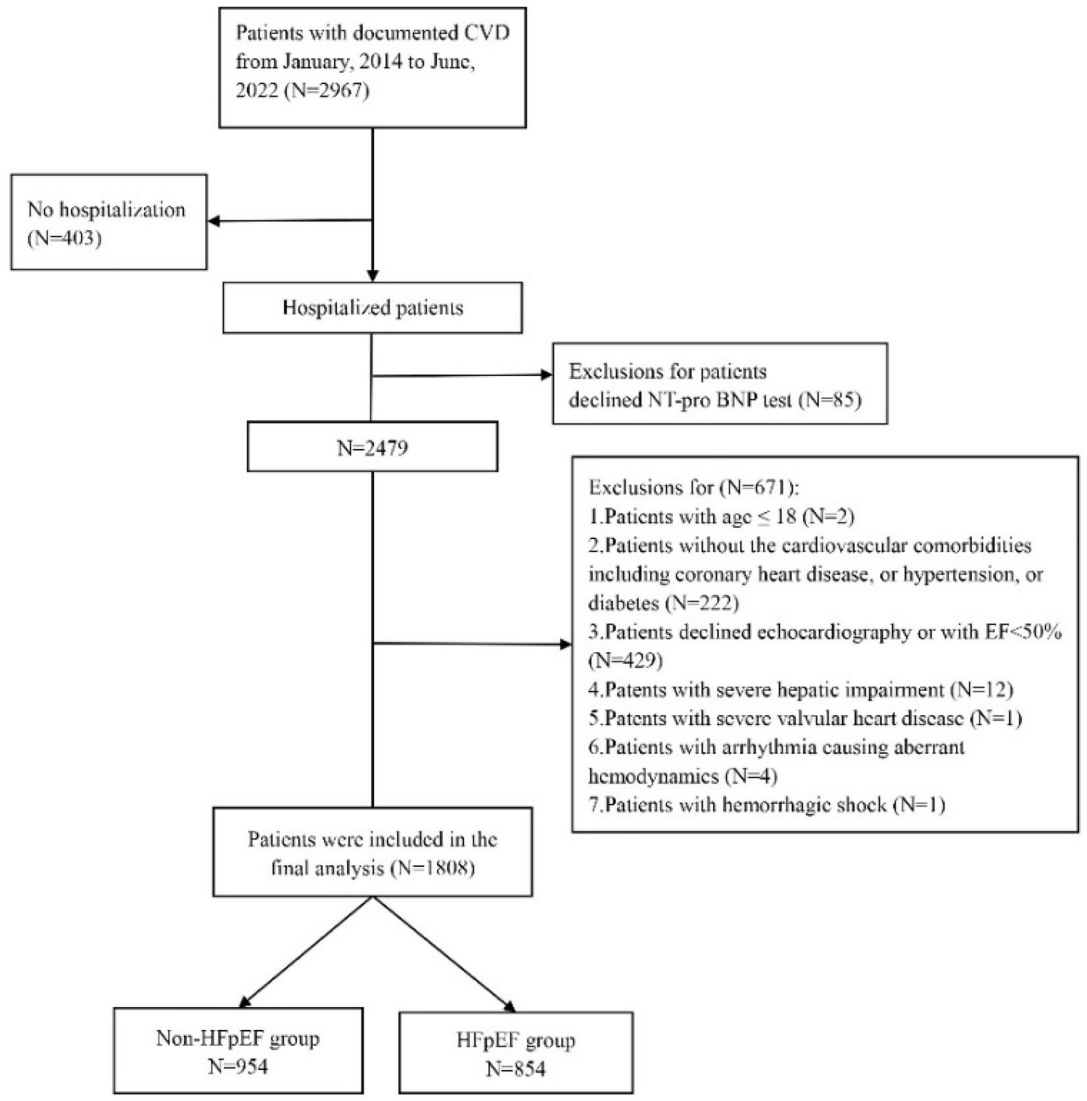
The flowchart of patient inclusion.

Figure 2 showed the flowchart of patient inclusion. A total of 1808 patients were analyzed in our study according to inclusion and exclusion criteria. Of these, 47.23% patients were HFpEF. Clinical characteristics of the HFpEF group and the non- HFpEF control were summarized in Table 1.

**Table 1:** Clinical characteristics of HFpEF vs. non-HFpEF patients.

Variables	Total subjects (n = 1808)	non-HFpEF patients (n = 954)	HFpEF patients (n = 854)	*p-*value
Demographics				
Age, yrs	68 ± 11	65 ± 11	71 ± 11	< 0.001
Female, n (%)	738 (40.8)	360 (37.7)	378 (44.3)	0.005
AF, n (%)	253 (14.0)	47 (4.9)	206 (24.1)	< 0.001
BMI, kg/m^2	27.44 ± 5.09	27.32 ± 4.95	27.59 ± 5.25	0.273
Smoking, n (%)	559 (30.9)	340 (35.6)	219 (25.6)	< 0.001
Alcohol habit, n (%)	432 (23.9)	270 (28.3)	162 (19.0)	< 0.001
Hypertension, n (%)	1424 (78.8)	726 (76.1)	698 (81.7)	0.003
Coronary heart diseases, n (%)	1297 (71.7)	684 (71.7)	613 (71.8)	0.969
Diabetes mellitus, n (%)	575 (31.8)	279 (29.3)	296 (34.7)	0.014
PH, n (%)	314 (17.4)	95 (10.0)	219 (25.6)	< 0.001
Laboratory Characteristics				
NT-proBNP, pg/ml	256 (100,594)	50 (20,162)	630 (407,1394)	< 0.001
WBC count, 10^9/L	6.50 ± 1.98	6.53 ± 1.87	6.46 ± 2.09	0.419
Lymphocyte count, 10^9/L	1.66 ± 0.63	1.75 ± 0.63	1.56 ± 0.62	< 0.001
Monocyte count, 10^9/L	0.44 ± 0.19	0.44 ± 0.18	0.44 ± 0.20	0.735
Neutrophil count, 10^9/L	4.23 ± 1.73	4.17 ± 1.61	4.29 ± 1.85	0.134
Eosinophil count, 10^9/L	0.10 (0.06,0.17)	0.10 (0.06,0.17)	0.10 (0.05,0.17)	0.22
Basophil count, 10^9/L	0.03 (0.02,0.04)	0.03 (0.02,0.05)	0.03 (0.02,0.04)	< 0.001
NLR	2.99 ± 2.41	2.71 ± 1.72	3.30 ± 2.98	< 0.001
Hb, g/L	130 ± 19	134 ± 16	125 ± 21	< 0.001
AST/ALT	1.21 ± 0.74	1.10 ± 0.47	1.34 ± 0.93	< 0.001
BUN, mmol/L	5.8 (4.8,7.2)	5.6 (4.7,6.6)	6.2 (4.9,8.3)	< 0.001
Cr, umol/L	74 (61,89)	71 (59,83)	78 (64,101)	< 0.001
UA, umol/L	340 ± 110	325 ± 101	357 ± 116	< 0.001
Total-cholesterol, mmol/L	3.95 ± 1.14	3.94 ± 1.04	3.95 ± 1.25	0.817
Triglycerides, mmol/L	1.62 ± 1.03	1.71 ± 1.11	1.51 ± 0.93	< 0.001
HDL-C, mmol/L	1.04 ± 0.25	1.04 ± 0.25	1.04 ± 0.25	0.885
LDL-C, mmol/L	2.23 ± 0.91	2.21 ± 0.79	2.26 ± 1.02	0.223
Apo A1, g/L	1.25 ± 0.23	1.27 ± 0.22	1.24 ± 0.23	0.033
Apo B, g/L	0.75 ± 0.25	0.75 ± 0.23	0.76 ± 0.28	0.894
Lpa, mg/L	178 (88,332)	162 (83,307)	200 (95,369)	< 0.001
Echocardiographic results				
LAD, mm	42 ± 6	40 ± 5	44 ± 7	< 0.001
LVDd, mm	49 ± 4	48 ± 4	49 ± 5	< 0.001
LVDs, mm	32 ± 3	31 ± 3	32 ± 4	< 0.001
LVPWD, mm	9 ± 1	9 ± 1	10 ± 1	< 0.001
IVSD, mm	10 ± 2	10 ± 2	11 ± 2	< 0.001
FS, %	35 ± 3	35 ± 2	34 ± 3	< 0.001
SV, ml	71 ± 15	70 ± 14	71 ± 16	0.14
LVEF, %	63 ± 3	64 ± 3	63 ± 4	< 0.001
Peak E velocity, cm/s	69 ± 19	69 ± 17	70 ± 21	0.161
Peak A velocity, cm/s	89 ± 19	87 ± 18	91 ± 21	< 0.001
Peak E' velocity, cm/s	6.0 ± 1.7	6.2 ± 1.7	5.7 ± 1.6	< 0.001
Peak A' velocity, cm/s	9.7 ± 2.0	9.9 ± 1.9	9.5 ± 2.1	0.001
E/A ratio	0.81 ± 0.30	0.82 ± 0.29	0.80 ± 0.32	0.397
E'/A' ratio	0.64 ± 0.20	0.64 ± 0.19	0.64 ± 0.21	0.811
E/E' ratio	12.35 ± 4.15	11.79 ± 3.64	13.14 ± 4.66	< 0.001

AF, atrial fibrillation; ALT, alanine aminotransferase; AST, aspartate aminotransferase; Apo A1, Apolipoproteins A1; Apo B, Apolipoproteins B; BUN, blood urea nitrogen; Cr, creatinine; FS, fractional shortening; Hb, hemoglobin; HDL-C, high-density lipoprotein cholesterol; IVSD, interventricular septal thickness; LDL-C, low-density lipoprotein cholesterol; Lpa, lipoprotein A; LAD, left atrial diameter; LVDd, left ventricular diameter in diastole; LVDs, left ventricular diameter in systole; LVPWD, End diastolic thickness of posterior left ventricular wall; LVEF, left ventricular ejection fraction; NT-proBNP, N-Terminal pro B-type natriuretic peptide; NLR, neutrophil to lymphocyte ratio; PH, pulmonary hypertension; Peak E, the maximum early transmitral flow velocity; Peak A, the maximum early transmitral flow velocity in atrial systole; Peak E', myocardial tissue velocity measured atthe septal and/or lateral mitral annulus; Peak A', the maximum myocardial tissue velocity measured at the mitral annulus in atrial systole; RBC, red blood cell; SV, stroke volume; UA, uric acid; WBC, white blood cell.

### Model development and presentation

We initially conducted univariate analysis to screen potential predictors for HFpEF in the 44 variables (*p* < 0.05). The univariate analysis revealed statistical differences in 8 variables in demographics (age, gender, AF, smoking, alcohol habit, hypertension, diabetes mellitus, and PH), 12 variables in laboratory parameters (NT-pro BNP, lymphocyte count, basophil count, NLR, Hb, AST/ALT ratio, BUN, Cr, UA, triglycerides, Apo A1 and Lpa), and 11 variables in echocardiographic results (LAD, LVDd, LVDs, LVPWD, IVSD, FS, LVEF, Peak A velocity, Peak E' velocity, Peak A' velocity, and E/E' ratio).

Next, echocardiographic indices and NT-pro BNP were part of the clinical diagnostic criteria for HFpEF. First, echocardiography and NT-pro BNP are not routinely performed in patients outside department of cardiology. Moreover, they are structural and functional alterations resultant from HFpEF other than causes or risks of HFpEF incidence. We excluded echocardiographic indices and NT-pro BNP when developing this prediction model. Then, 19 variables were selected as candidate predictors, including age, gender, AF, smoking, alcohol habit, hypertension, diabetes mellitus, PH, lymphocyte count, basophil count, NLR, Hb, AST/ALT ratio, BUN, Cr, UA, triglycerides, Apo A1, and Lpa. By multivariate regression analysis, 9 variables were selected as significant predictors of HFpEF (Table 2). By LASSO regression analysis, 9 variables were selected, including Age, L, Hb, NLR, AST/ALT, Cr, UA, AF, PH. After taking the intersection, 8 variables were obtained, namely optimal predictors for HFpEF, and a final model was built using the logistic regression model (Table 3). In this model, AF was an independent predictor with a 4.7-fold increased risk for HFpEF (OR, 4.70; 95% CI 3.32–6.77; *p* < 0.001).

**Table 2:** Multivariable analyses of candidate predictors for HFpEF.

Variables	OR	OR 95% CI	*p-*value
Age, yrs	1.02	(1.01,1.03)	< 0.001
Hb, g/L	0.985	(0.98,0.99)	< 0.001
NLR	1.082	(1.02,1.14)	0.006
AST/ALT	1.619	(1.30,2.02)	< 0.001
BUN, mmol/L	0.985	(0.97,1.00)	0.052
Cr, umol/L	1.003	(1.00,1.01)	< 0.001
UA, umol/L	1.003	(1.00,1.01)	< 0.001
AF, n(%)	4.718	(3.30,6.74)	< 0.001
PH, n(%)	1.837	(1.37,2.47)	< 0.001

ALT, alanine aminotransferase; AST, aspartate aminotransferase; AF, atrial fibrillation; BUN, blood urea nitrogen; Cr, creatinine; Hb, Hemoglobin; NLR, neutrophil to lymphocyte ratio; PH, pulmonary hypertension; UA, uric acid.

**Table 3:** The predictors of HFpEF.

Variables	OR	OR 95% CI	*p-*value
Age, yrs	1.02	1.010,1.031	< 0.001
Hb, g/L	0.98	0.978,0.991	< 0.001
NLR	1.07	1.017,1.134	0.014
AST/ALT	1.58	1.277,1.979	< 0.001
Cr, umol/L	1.00	1.001,1.005	< 0.001
UA, umol/L	1.00	1.000,1.003	0.004
AF, n(%)	4.70	3.319,6.765	< 0.001
PH, n(%)	1.82	1.360,2.449	< 0.001

ALT, alanine aminotransferase; AST, aspartate aminotransferase; AF, atrial fibrillation; Cr, creatinine; Hb, Hemoglobin; NLR, neutrophil to lymphocyte ratio; PH, pulmonary hypertension; UA, uric acid.

### Model evaluation and validation

**Figure 3:**
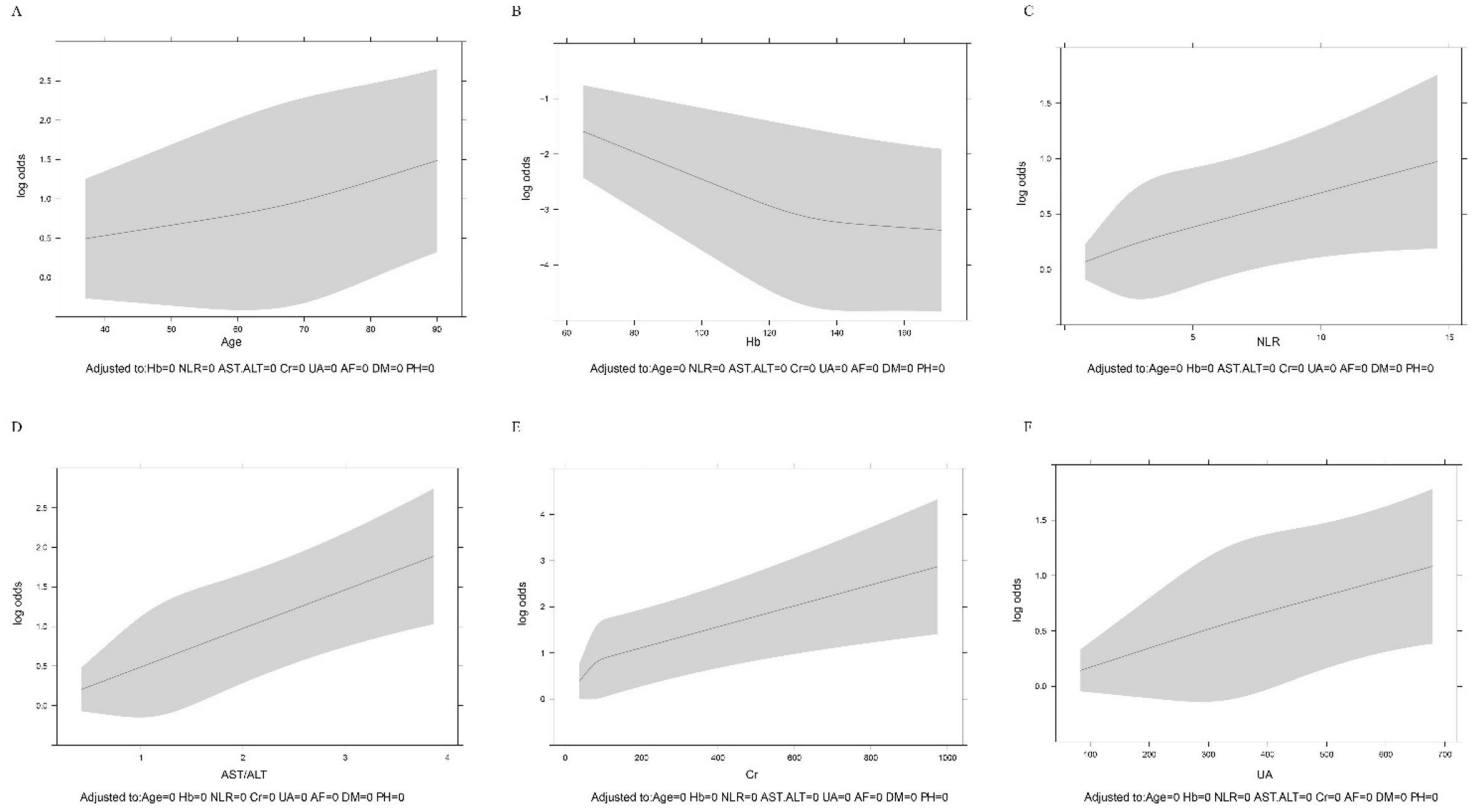
Linear relationship diagram (**A**, **B**, **C**, **D**, **E** and **F**). (**A**) Linear relationship between age and HFpEF. (**B**) Linear relationship between Hb and HFpEF. (**C**) Linear relationship between NLR and HFpEF. (**D**) Linear relationship between AST/ALT ratio and HFpEF. (**E**) Linear relationship between Cr and HFpEF. (**F**) Linear relationship between UA and HFpEF.

No significant multicollinearity was observed for any variables included in our model (Table 4). The potential linear relationship between each variable and HFpEF was assessed by the spline function (Fig. 3). There was a positive linear relationship between age, NLR, UA, AST/ALT ratio, Cr and HFpEF, and there was an inversely linear relationship between Hb and HFpEF.

**Table 4:** The multicollinearity of variables in the model.

Variables	Multicollinearity
Age, yrs	1.15
Hb, g/L	1.19
NLR	1.03
AST/ALT	1.10
Cr, umol/L	1.14
UA, umol/L	1.08
AF, n(%)	1.06
PH, n(%)	1.06

ALT, alanine aminotransferase; AST, aspartate aminotransferase; AF, atrial fibrillation; Cr, creatinine; Hb, Hemoglobin; NLR, neutrophil to lymphocyte ratio; PH, pulmonary hypertension; UA, uric acid.

**Figure 4:**
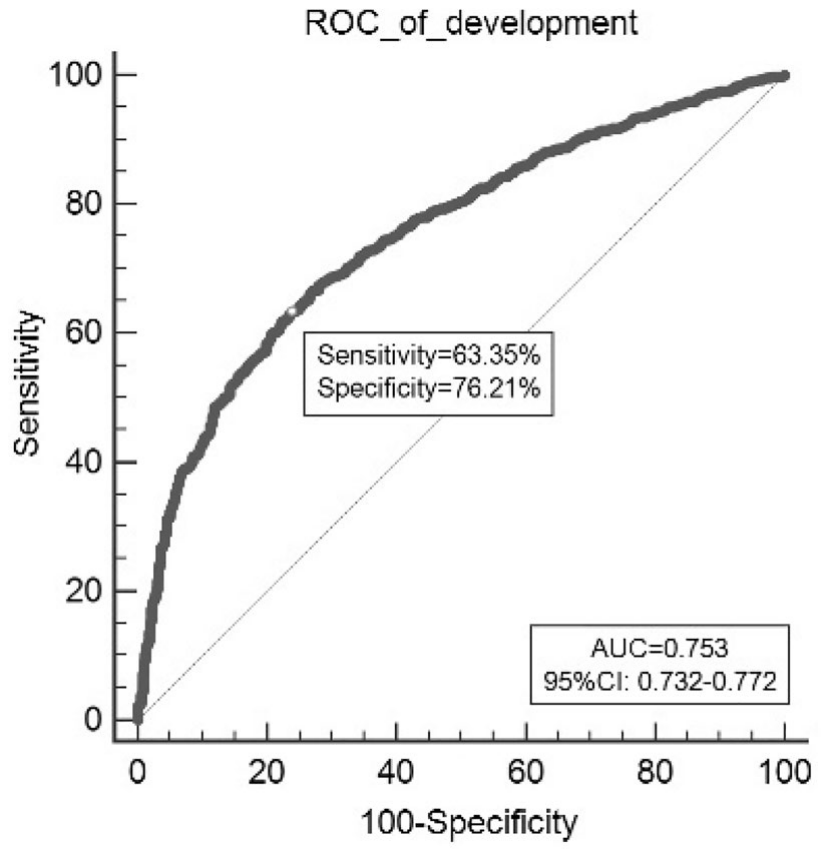
Receiver operating characteristic curves (ROC) of model in the development data.

**Figure 5:**
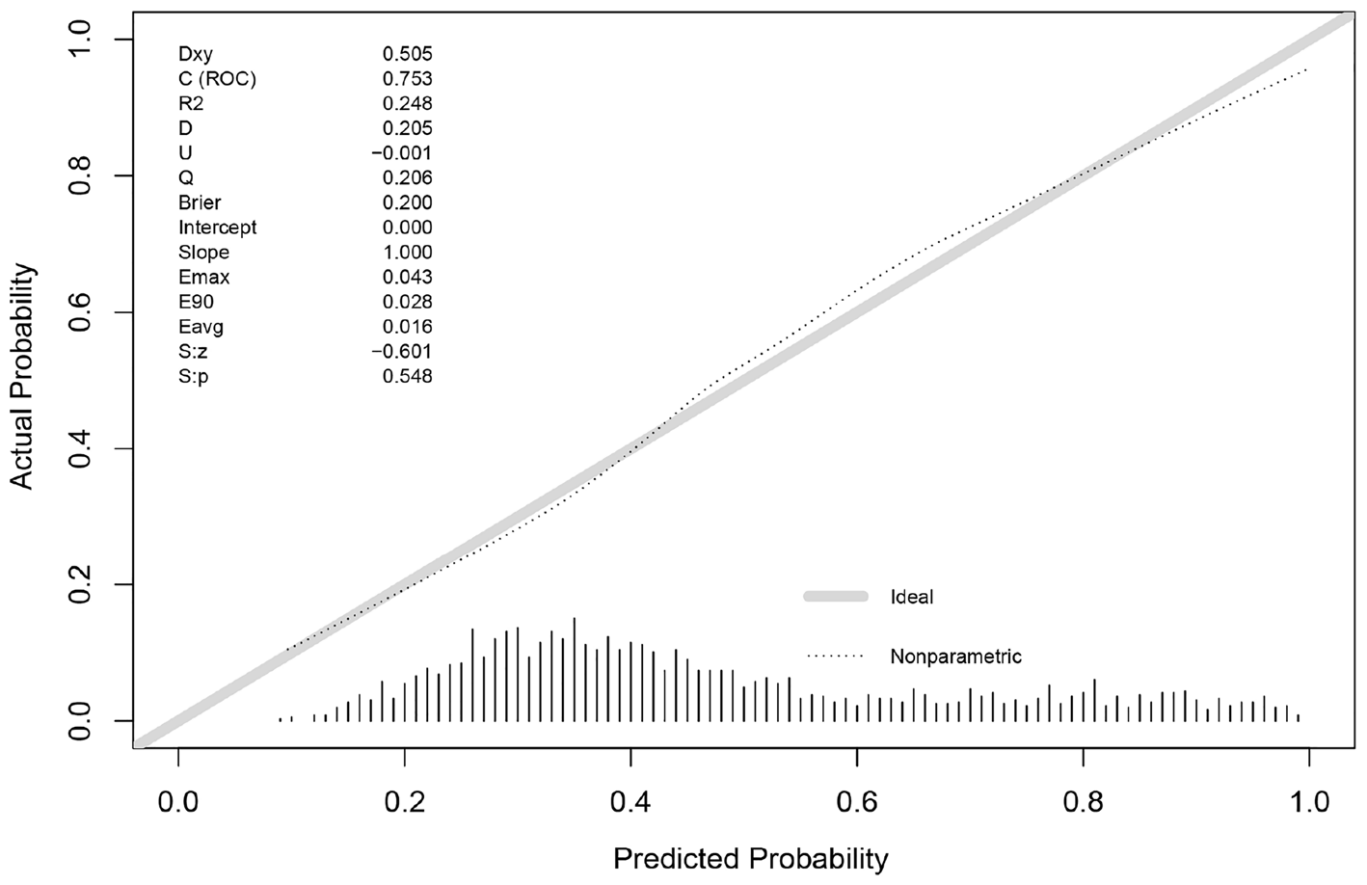
Calibration plot for model-predicted probability of HFpEF in the development data.

**Figure 6:**
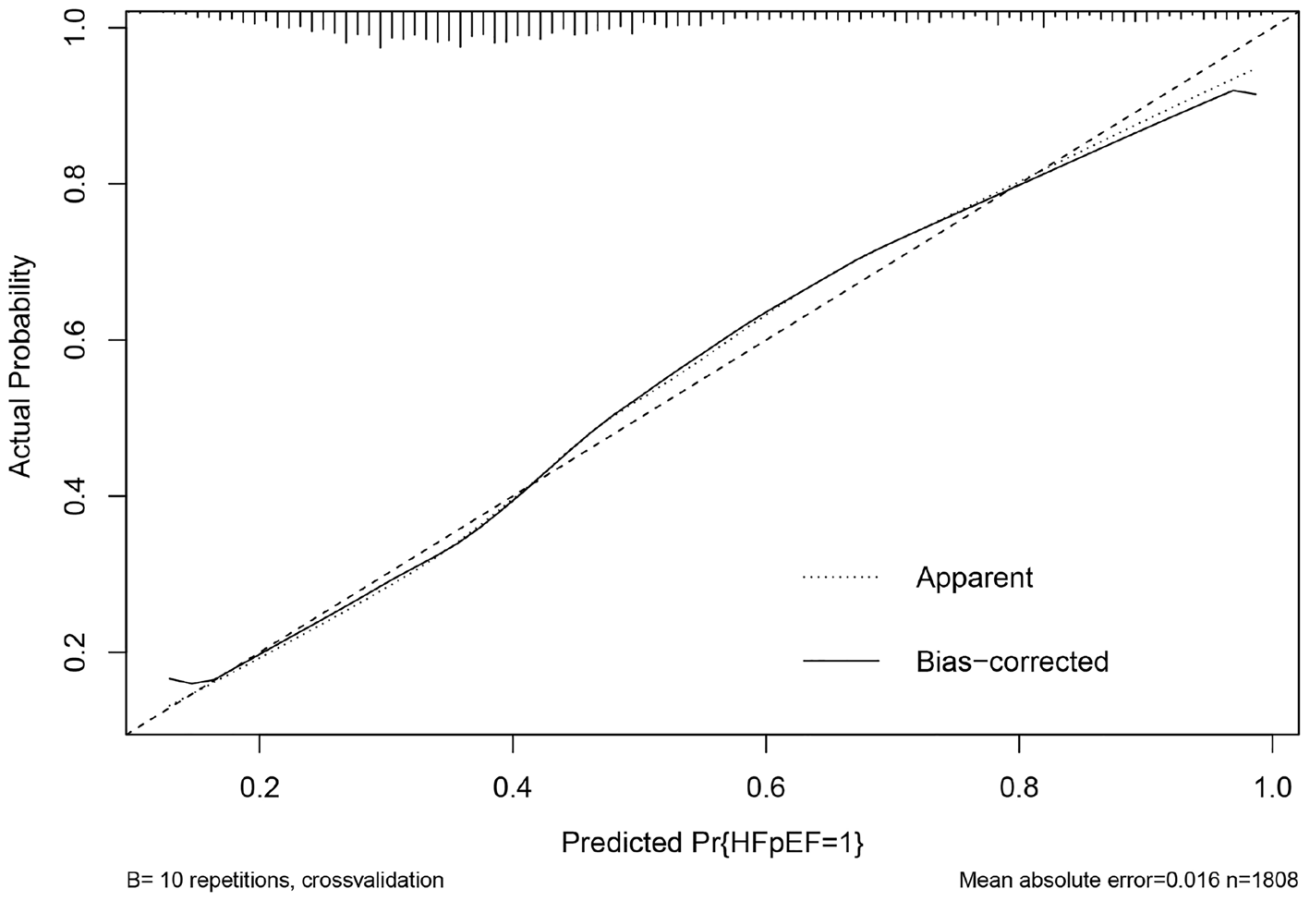
Tenfold cross-validated calibration plot.

The ROC of this model was 0.753 (95% CI 0.732–0.772) (Fig. 4), indicating the satisfactory discrimination. The Youden index, sensitivity, and specificity were 0.396, 63.35% and 76.21%, respectively. The optimal cutoff of predicted probability was 0.451. The patient was considered to have HFpEF when the predicted probability was greater than 0.451. The Brier score was 0.200, and the calibration plots showed favorable consistency between the prediction of the model and actual observations (Fig. 5). The internal validation showed remarkable discrimination and calibration (AUC = 0.750, Brier score = 0.202) (Table 5). The tenfold cross-validated calibration plot with good stability and accuracy was shown in Fig. 6.

**Table 5:** The diagnostic performance of model.

Variables	AUC	Brier score
Development cohort	0.753	0.200
Internal validation	0.750	0.202

**Figure 7:**
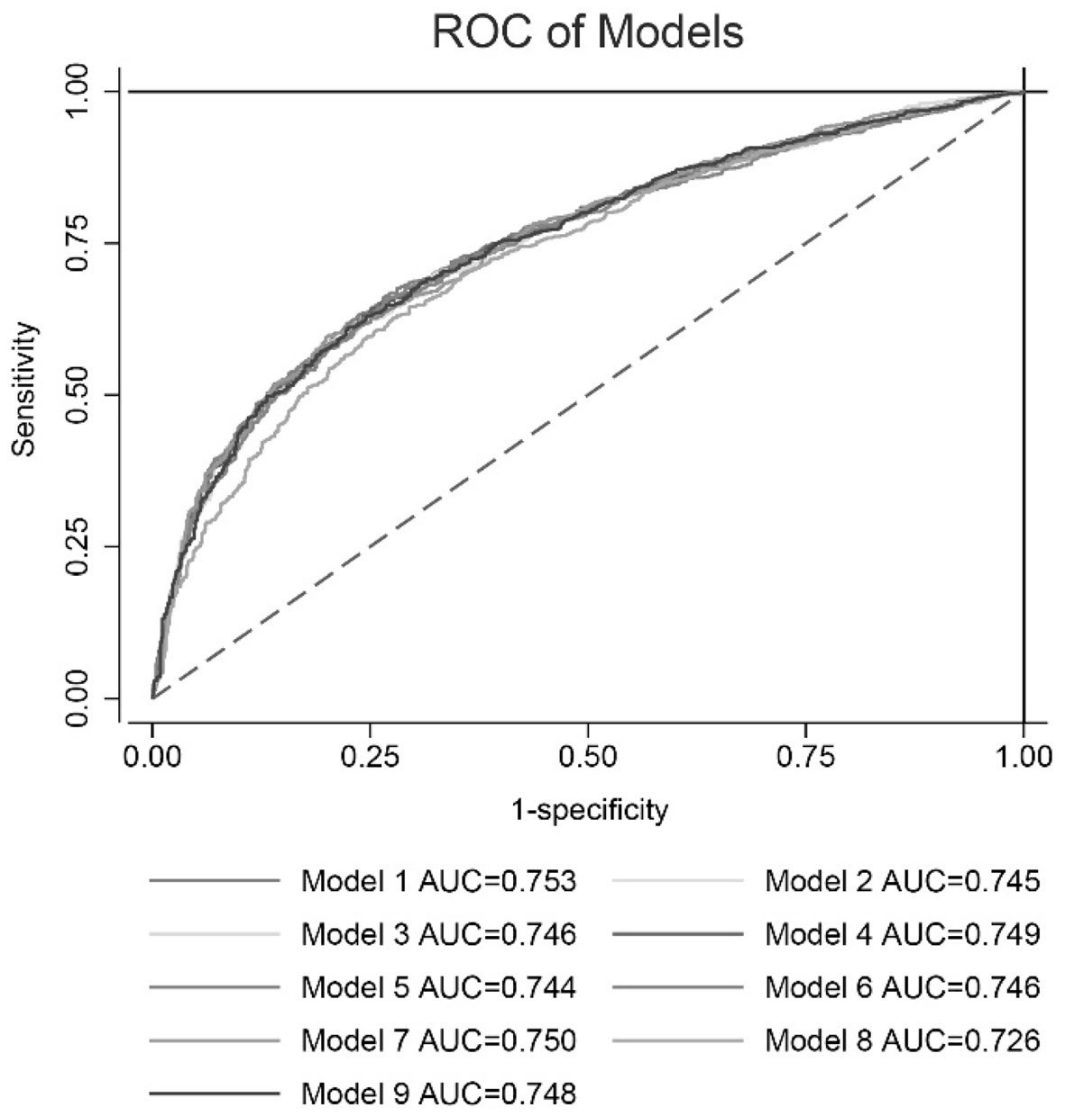
The ROC of different models.

**Figure 8:**
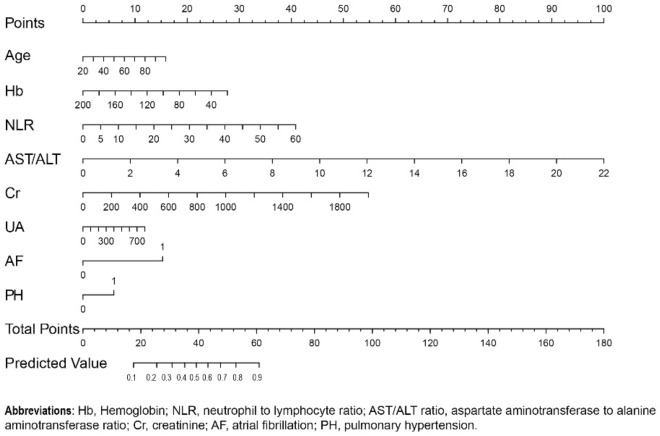
The nomogram of the model for predicting the probability of HFpEF.

We rebuilt the model by removing any one predictor, and calculated the predictive ability of each new model (Model 2-Model 9). The ROC of different models was presented as Fig. 7. We found the additive effects of AF, AST/ALT ratio and Cr on predictive of model by Z test (Table 6). In addition, through likelihood ratio test, the original model showed a higher goodness of fit compared with each new model (Table 7). The prediction nomogram for HFpEF was constructed, comprising age, Hb, NLR, AST/ALT ratio, Cr, UA, AF, and PH (Fig. 8). The weighted score was assigned to each of the independent predictors, and a higher score calculated from the sum of the assigned points for each predictor corresponding to a higher probability of HFpEF. In this nomogram, the highest total score was 100 points, and the scale of predicted value of HFpEF ranged from 0.1 to 0.9. Moreover, we designed a web-based online risk calculator, and made it freely available to all user (https://rose619.shinyapps.io/dynnomapp/). After entering related variables in the interface, we can get participant’s probability of HFpEF.

**Table 6:** The comparison of performance in different models.

Model	Age	Hb	NLR	AST/ALT	Cr	UA	AF	PH	AUC	*Z*	*p-*value
Model 1	+	+	+	+	+	+	+	+	0.753		
Model 2	–	+	+	+	+	+	+	+	0.745	2.100	0.036
Model 3	+	–	+	+	+	+	+	+	0.746	1.774	0.076
Model 4	+	+	–	+	+	+	+	+	0.749	1.583	0.114
Model 5	+	+	+	–	+	+	+	+	0.744	2.547	0.011
Model 6	+	+	+	+	–	+	+	+	0.746	3.233	0.001
Model 7	+	+	+	+	+	–	+	+	0.750	1.069	0.285
Model 8	+	+	+	+	+	+	–	+	0.726	4.892	< 0.001
Model 9	+	+	+	+	+	+	+	–	0.748	1.531	0.126

ALT, alanine aminotransferase; AST, aspartate aminotransferase; AF, atrial fibrillation; Cr, creatinine; Hb, Hemoglobin; NLR, neutrophil to lymphocyte ratio; PH, pulmonary hypertension; UA, uric acid.

**Table 7:** The likelihood ratio test for different models.

Model	Age	Hb	NLR	AST/ALT	Cr	UA	AF	PH	χ^2^	*p-*value
Model 1	+	+	+	+	+	+	+	+		
Model 2	–	+	+	+	+	+	+	+	14.88	< 0.001
Model 3	+	–	+	+	+	+	+	+	21.94	< 0.001
Model 4	+	+	–	+	+	+	+	+	7.19	0.007
Model 5	+	+	+	–	+	+	+	+	17.98	< 0.001
Model 6	+	+	+	+	–	+	+	+	19.28	< 0.001
Model 7	+	+	+	+	+	–	+	+	8.54	0.003
Model 8	+	+	+	+	+	+	–	+	84.66	< 0.001
Model 9	+	+	+	+	+	+	+	–	16.25	< 0.001

Hb, Hemoglobin; NLR, neutrophil to lymphocyte ratio; ALT, alanine aminotransferase; AST, aspartate aminotransferase; Cr, creatinine; UA, uric acid; AF, atrial fibrillation; PH, pulmonary hypertension.

## Discussion

In this study, we developed and validated a novel diagnostic prediction model for HFpEF using the clinical data from ChiHFpEF cohort. The model was built with eight predictors of HFpEF, including age, Hb, NLR, AST/ALT ratio, Cr, UA, AF, and PH. This model showed excellent discrimination, calibration, and well internal validation. Of note, to emphasize the importance of inflammation and organ interaction in the pathogenesis of HFpEF, this model incorporated the inflammatory markers and markers for liver and renal function. We also excluded clinical diagnostic criteria for HFpEF including echocardiographic indices and NT-pro BNP because they were used in the adjudication of HFpEF diagnosis. Therefore, this new score can be applicable in hospital departments without results of echocardiography and natriuretic peptides examination comparing with previous diagnostic scoring systems for HFpEF.

HFpEF was considered as a syndrome recapitulating the proinflammatory state^14^. In cardiac specimens of both HFpEF animals and patients, it was observed that inflammatory cell infiltration was associated with cardiac inflammation and fibrotic damages^33–35^. The proinflammatory molecules could lead to cardiomyocyte hypertrophy and passive stiffness through increasing oxidative stress and reducing nitric oxide bioavailability, and it could also cause cardiac interstitial fibrosis by triggering abnormal extracellular matrix conversion^14,36,37^. In the mouse model, the interaction between inflammation and mitochondrial hyperacetylation was thought to be a key driver in HFpEF pathogenesis, which could be ameliorated by enhancing β-hydroxybutyrate abundance^38^. In this study, we found that NLR was independently associated with the HFpEF, consistent to the report that NLR might be useful to stratify the risk of patients hospitalized with HFpEF^39^. Moreover, previous studies have revealed elevated UA could lead to inflammation and oxidative stress in vascular endothelial cells^20^. UA was an independent predictor of adverse outcomes in patients with HFpEF^19–21,40^, and lowering UA might have a beneficial effect on the prognosis of patients with hyperuricemia and HFpEF^20^. In this study, we found the elevation of UA was a diagnostic predictor for HFpEF, suggesting that UA may be a relevant target for clinical prevention and treatment in HFpEF. Therefore, introducing these inflammatory markers into the diagnostic model may improve the prediction efficiency, and facilitate the identification of patients at different risk for HFpEF.

HFpEF was generally recognized as a disease of elderly, with the majority of patients age > 65 years^41^. In the patients with HFpEF, the PH was highly prevalent^42^. The pulmonary venous congested passively in the setting of elevated left atrium pressure, and pulmonary arterial vessels constricted or remodeled, resulting in the post-capillary PH, or the combined pre-capillary and post-capillary PH^42,43^. PH was considered as a critical prognostic factor for HFpEF^42^. Our study indicated that PH was one of predictors of HFpEF, suggesting the improvement of pulmonary vascular function may be a potential therapeutic target for HFpEF. In addition, we found AF was a major predictor for HFpEF, and Reddy’s study also demonstrated that AF increased significantly risk for HFpEF^8,44^. AF could directly cause the development of HFpEF by remodeling left atrium, impairing atrial function, and aggravating atrial fibrosis^45^. Considering catheter ablation plays an important role in the treatment of AF, further studies are needed to explore whether catheter ablation could reduce the incidence of HFpEF in the future.

A heart-liver axis, that was drafted by inflammatory reactants from the heart and the liver, placed a new spotlight on the crosstalk of organs in disease development^23,46^. In the phenomapping analysis of HFpEF, one of phenogroups demonstrated high proinflammatory biomarkers including tumor necrosis factor-alpha-mediated inflammation, liver fibrosis, and tissue remodeling^47^. Chirinos’ study indicated these biomarker clusters consisting of fibrosis/tissue remodeling, inflammation, and liver fibrosis were correlated with the outcomes of HFpEF^48^. In addition, AST/ALT ratio was used as one of predictors of liver fibrosis^49^, and a prospective observational study demonstrated that the non-alcoholic fatty liver disease fibrosis score, based on AST/ALT ratio, platelet counts, and albumin, was associated with higher all-cause mortality in HFpEF patients^50^. We found the AST/ALT ratio was a predictor for HFpEF, suggesting the correlation between liver fibrosis and HFpEF. Recently, Lusis' group found that a liver-derived protein, coagulation factor XI, could protect against diastolic dysfunction and decrease the cardiac fibrosis and inflammation, which was associated with the activity of SMAD pathway^51^. Therefore, identifying the mediator of liver-heart cross-talk and focusing on heart-liver axis may stand for the new target for the treatment of HFpEF.

The bidirectional interaction between the failing heart and kidneys was described as the cardiorenal syndrome^52^. Electrolyte dysregulation and fluid retention were common in patients with HFpEF, and renal insufficiency was thought as the main reason^53^. Previous studies indicated that renal insufficiency was a major predictor of outcome for HFpEF^53,54^, and we also found elevated Cr was associated with increasing risk of HFpEF. There were overlapping pathophysiological changes including systemic inflammation, oxidative stress, arterial stiffening, and endothelial dysfunction between HFpEF and renal insufficiency^53,55^. These pathways contribute to cardiac diastolic dysfunction, nephron loss, nephron compensatory hypertrophy and hyperfiltration of the remaining nephrons^53^. Therefore, focusing on the alleviation of factors provoking renal injury and slowing the progression of renal dysfunction may be helpful to reduce the risk of HFpEF.

Anemia was considered as an independent risk factor for the pathogenesis and development of HFpEF^56,57^. Similarly, we found a negative linear relationship between Hb with HFpEF. Anemia could reduce oxygen-carrying capacity of the blood, and increase the myocardial work, leading to the disbalance between myocardial oxygen demand and supply^57^. The deficiency of iron was the commonest contributing factor to anemia in HF patients^58^. In a double-blind randomized controlled trial, the HF patients with iron deficiency had significant improvements in functional capacity and quality of life when given intravenously ferric carboxymaltose^59^. Therefore, improving the Hb for patients with anemia might reduce the risk of HFpEF.

Several limitations should be considered. First, this study was conducted in only single heart center, and the sample size was relatively small. Owing to the study population was only patients with coronary heart disease, or hypertension, or diabetes mellitus in hospital, selection bias was inevitable in this study. Second, although the model presented favorable discrimination and calibration ability in the development data and internal validation, the validation with Chinese Han population or with existing data sets or in other centers was not performed, which may limit the generalizability of model.

## Conclusions

In this study, we developed and validated a novel, simple model with inflammatory markers and markers for liver and renal dysfunction to predict the probability of HFpEF in patients with CVDs. This diagnostic model could improve the clinical prediction efficiency and facilitate the identification of patients at different risk for HFpEF.

## Data Availability

The datasets used and/or analyzed during the current study are available from the corresponding author upon reasonable request.
